# EM Algorithm for Mapping Quantitative Trait Loci in Multivalent Tetraploids

**DOI:** 10.1155/2010/216547

**Published:** 2011-01-05

**Authors:** Jiahan Li, Kiranmoy Das, Guifang Fu, Chunfa Tong, Yao Li, Christian Tobias, Rongling Wu

**Affiliations:** ^1^Center for Statistical Genetics, Pennsylvania State University, Hershey, PA 17033, USA; ^2^Department of Statistics, West Virginia University, Morgantown, WV 26506, USA; ^3^Genomics and Gene Discovery Research Unit, USDA-ARS Western Regional Research Center, Albany, CA 94710, USA

## Abstract

Multivalent tetraploids that include many plant species, such as potato, sugarcane, and rose, are of paramount importance to agricultural production and biological research. Quantitative trait locus (QTL) mapping in multivalent tetraploids is challenged by their unique cytogenetic properties, such as double reduction. We develop a statistical method for mapping multivalent tetraploid QTLs by considering these cytogenetic properties. This method is built in the mixture model-based framework and implemented with the EM algorithm. The method allows the simultaneous estimation of QTL positions, QTL effects, the chromosomal pairing factor, and the degree of double reduction as well as the assessment of the estimation precision of these parameters. We used simulated data to examine the statistical properties of the method and validate its utilization. The new method and its software will provide a useful tool for QTL mapping in multivalent tetraploids that undergo double reduction.

## 1. Introduction

 Genetic analysis in polyploids has received considerable interest in recent years because of the biological and economic importance [1–3]. Genetic linkage maps constructed from molecular markers have been published for several major polyploids [4–10]. Statistical models for linkage analysis and map construction that consider unique biological properties of polyploids have been developed [11–14]. For bivalent polyploids, Wu et al. [15, 16] incorporated the so-called chromosomal pairing preference [17] into the linkage analysis framework, to increase the biological relevance of linkage mapping models. There have been several statistical models developed to map quantitative trait loci (QTLs) in bivalent polyploids [18, 19].

There is also a group of polyploids, called multivalent polyploids, in which chromosomes pair among more than two homologous copies at meiosis, rather than only two copies as like in bivalent polyploids. The origin of multivalent polyploids is mostly from the duplication of similar genomes and, for this reason, they are called autopolyploids [20, 21]. The consequence of multivalent pairing in autopolyploids is the occurrence of double reduction, that is, two sister chromatids of a chromosome sort into the same gamete [22]. Fisher [23] proposed a conceptual model for characterizing the individual probabilities of 11 different modes of gamete formation for a quadrivalent polyploid in terms of the recombination fraction between two different loci and their double reductions. Wu et al. [24] used Fisher's model to derive the EM algorithm for the estimation of the linkage between fully informative markers. Wu and Ma [25] extended this model to analyze any type of markers, regardless of their informativeness and dominant or codominant nature. The significant advantage of the models by Wu and colleagues directly lies in their generality, flexibility, and robustness.

In this paper, we develop a statistical method for QTL mapping in multivalent tetraploids by considering Fisher's [23] 11 classifications of gamete formation. The method allows the estimation and test of not only the QTL-marker linkage, but also the extent of double reduction of the QTL. Because of the inherent complexity of classification analyses of gamete formation, we will focus on the modeling and analysis of one-marker/one-QTL associations. A two-stage hierarchical model is derived to estimate the probabilities of gamete formation modes and therefore double reduction in the upper hierarchy and estimate the marker-QTL recombination fraction in the lower hierarchy within the maximum likelihood context implemented with the EM algorithm. The method is used to analyze a simulated data set, with results demonstrating statistical properties of the method and its analytical and biological merits.

## 2. Method

### 2.1. Genetic Design

Consider a heterozygous multivalent tetraploid line crossed with a homozygous line to generate a so-called pseudotest backcross population. For such a population, the genotypes of progeny are consistent with the genotypes of gametes produced by the heterozygous parent and, therefore, the derivation of mapping models can be based on the segregation of gametes. Suppose there are *n* individuals in the pseudotest backcross population. A panel of codominant markers is typed for each individual, with which a linkage map is constructed. All the pseudotest backcross individuals are phenotyped for a quantitative trait that is assumed to be controlled by QTLs.

### 2.2. Tetrasomic Co-Inheritance

To simplify our analysis, we assume that the QTLs underlying the trait are mapped with single markers. Let *M*_1_,…, *M*_4_ be the four alleles at a marker **M**, and let *Q*_1_,…, *Q*_4_ be the four alleles at a QTL linked with the marker. The marker and QTL are linked with a recombination fraction of *r*. Because of the double reduction, the multivalent tetraploid generates 10 diploid gametes for locus, which are arrayed as (*M*_1_ *M*_1_, *M*_2_ *M*_2_, *M*_3_ *M*_3_, *M*_4_ *M*_4_, *M*_1_ *M*_2_, *M*_1_ *M*_3_, *M*_1_ *M*_4_, *M*_2_ *M*_3_, *M*_2_ *M*_4_, *M*_3_ *M*_4_) for the markers, and (*Q*_1_ *Q*_1_, *Q*_2_ *Q*_2_, *Q*_3_ *Q*_3_, *Q*_4_ *Q*_4_, *Q*_1_ *Q*_2_, *Q*_1_ *Q*_3_, *Q*_1_ *Q*_4_, *Q*_2_ *Q*_3_, *Q*_2_ *Q*_4_, *Q*_3_ *Q*_4_) for the QTL. In each case, the first four gametes are derived from the double reduction, whereas the second six gametes are derived from the chromosome paring. Let  *α* and  *β* be the frequencies of double reduction at the marker and QTL, respectively. The frequency of double reduction is a constant for any given locus, with the value depending on its distance from the centromere.

When the marker and QTL are co-segregating in a multivalent tetraploid, a total of 136 diploid gamete formation mechanisms are generated although there are only 100 gamete genotypes that are observable. Based on the presence/absence of double reduction and the number of recombinant events, Fisher [23] classified these 136 formation mechanisms into 11 gamete modes. Of these 11 gamete modes, however, only nine can be observed each with a frequency denoted by *g*_*h*_  (*h* = 1,…, 9). These 9 observable gamete modes were rearranged by Wu et al. [24] in matrix form expressed as

(1) $$\begin{array}{cc} & \begin{array}{cccccccccccc} Q_{1}Q_{1} & Q_{2}Q_{2} & Q_{3}Q_{3} & Q_{4}Q_{4} & Q_{1}Q_{2} & Q_{1}Q_{3} & Q_{1}Q_{4} & Q_{2}Q_{3} & Q_{2}Q_{4} & Q_{3}Q_{4} & & \end{array}\\ \begin{array}{c} \;M_{1}M_{1}\\ \;M_{2}M_{2}\\ \;M_{3}M_{3}\\ \;M_{4}M_{4}\\ g=\frac{M_{1}M_{2}}{M_{2}M_{1}}\\ \;\frac{M_{1}M_{3}}{M_{3}M_{1}}\\ \;\frac{M_{1}M_{4}}{M_{4}M_{1}}\\ \;\frac{M_{2}M_{3}}{M_{3}M_{2}}\\ \;\frac{M_{2}M_{4}}{M_{4}M_{2}}\\ \;\frac{M_{3}M_{4}}{M_{4}M_{3}} \end{array} & \left[\begin{array}{cccccccccc} \frac{1}{4}g_{1} & \frac{1}{12}g_{2} & \frac{1}{12}g_{2} & \frac{1}{12}g_{2} & \frac{1}{12}g_{5} & \frac{1}{12}g_{5} & \frac{1}{12}g_{5} & \frac{1}{12}g_{6} & \frac{1}{12}g_{6} & \frac{1}{12}g_{6}\\ \frac{1}{12}g_{2} & \frac{1}{4}g_{1} & \frac{1}{12}g_{2} & \frac{1}{12}g_{2} & \frac{1}{12}g_{5} & \frac{1}{12}g_{6} & \frac{1}{12}g_{6} & \frac{1}{12}g_{5} & \frac{1}{12}g_{5} & \frac{1}{12}g_{6}\\ \frac{1}{12}g_{2} & \frac{1}{12}g_{2} & \frac{1}{4}g_{1} & \frac{1}{12}g_{2} & \frac{1}{12}g_{6} & \frac{1}{12}g_{5} & \frac{1}{12}g_{6} & \frac{1}{12}g_{5} & \frac{1}{12}g_{6} & \frac{1}{12}g_{5}\\ \frac{1}{12}g_{2} & \frac{1}{12}g_{2} & \frac{1}{12}g_{2} & \frac{1}{4}g_{1} & \frac{1}{12}g_{6} & \frac{1}{12}g_{6} & \frac{1}{12}g_{5} & \frac{1}{12}g_{6} & \frac{1}{12}g_{5} & \frac{1}{12}g_{5}\\ \frac{1}{12}g_{3} & \frac{1}{12}g_{3} & \frac{1}{12}g_{4} & \frac{1}{12}g_{4} & \frac{1}{6}g_{7} & \frac{1}{24}g_{8} & \frac{1}{24}g_{8} & \frac{1}{24}g_{8} & \frac{1}{24}g_{8} & \frac{1}{6}g_{9}\\ \frac{1}{12}g_{3} & \frac{1}{12}g_{4} & \frac{1}{12}g_{3} & \frac{1}{12}g_{4} & \frac{1}{24}g_{8} & \frac{1}{6}g_{7} & \frac{1}{24}g_{8} & \frac{1}{24}g_{8} & \frac{1}{6}g_{9} & \frac{1}{24}g_{8}\\ \frac{1}{12}g_{3} & \frac{1}{12}g_{4} & \frac{1}{12}g_{4} & \frac{1}{12}g_{3} & \frac{1}{24}g_{8} & \frac{1}{24}g_{8} & \frac{1}{6}g_{7} & \frac{1}{6}g_{9} & \frac{1}{24}g_{8} & \frac{1}{24}g_{8}\\ \frac{1}{12}g_{4} & \frac{1}{12}g_{3} & \frac{1}{12}g_{3} & \frac{1}{12}g_{4} & \frac{1}{24}g_{8} & \frac{1}{24}g_{8} & \frac{1}{6}g_{9} & \frac{1}{6}g_{7} & \frac{1}{24}g_{8} & \frac{1}{24}g_{8}\\ \frac{1}{12}g_{4} & \frac{1}{12}g_{3} & \frac{1}{12}g_{4} & \frac{1}{12}g_{3} & \frac{1}{24}g_{8} & \frac{1}{6}g_{9} & \frac{1}{24}g_{8} & \frac{1}{24}g_{8} & \frac{1}{6}g_{7} & \frac{1}{24}g_{8}\\ \frac{1}{12}g_{4} & \frac{1}{12}g_{4} & \frac{1}{12}g_{3} & \frac{1}{12}g_{3} & \frac{1}{6}g_{9} & \frac{1}{24}g_{8} & \frac{1}{24}g_{8} & \frac{1}{24}g_{8} & \frac{1}{24}g_{8} & \frac{1}{6}g_{7} \end{array}\right], \end{array}$$

where *g*_1_ and *g*_2_ are associated with double reductions at both the marker and QTL, *g*_3_ and *g*_4_ with double reductions only at QTL **Q**, *g*_5_ and *g*_6_ with double reductions only at marker **M**, and *g*_7_ − *g*_9_ with nondouble reductions. From matrix (1), we see that there are no, one and two recombinant events in the cells (*g*_1_), (*g*_3_, *g*_5_), and (*g*_2_, *g*_4_, *g*_6_, *g*_9_), respectively. The cells (*g*_7_) and (*g*_8_) are each a mixture of two different gamete formation mechanisms or configurations (*A* and *B*), that is, *g*_7_ = *g*_7*A*_ + *g*_7*B*_ and *g*_8_ = *g*_8*A*_ + *g*_8*B*_, with relative proportions determined by *r*. Because different configurations contain different numbers of recombination events, the expected number of recombination events in each cell, that is, an observable gamete genotype, should be the weighted average of the number of recombination events for each configuration. Wu et al. [24] used a matrix form (**e**) to count the expected number of recombination events for each observable gamete genotype expressed as

(2) $$\begin{array}{cc} c= & \\ & \begin{array}{cccccccccc} Q_{1}Q_{1}\;\; & Q_{2}Q_{2}\; & Q_{3}Q_{3}\; & Q_{4}Q_{4}\; & \;\;Q_{1}Q_{2}\; & \;\;Q_{1}Q_{3}\; & \;\;Q_{1}Q_{4}\; & \;\;Q_{2}Q_{3} & \;\;Q_{2}Q_{4}\; & \;\;Q_{3}Q_{4}\; \end{array}\\ \begin{array}{c} M_{1}M_{1}\\ M_{2}M_{2}\\ M_{3}M_{3}\\ M_{4}M_{4}\\ \frac{M_{1}M_{2}}{M_{2}M_{1}}\\ \frac{M_{1}M_{3}}{M_{3}M_{1}}\\ \frac{M_{1}M_{4}}{M_{4}M_{1}}\\ \frac{M_{2}M_{3}}{M_{3}M_{2}}\\ \frac{M_{2}M_{4}}{M_{4}M_{2}}\\ \frac{M_{3}M_{4}}{M_{4}M_{3}} \end{array} & \left[\begin{array}{cccccccccc} 0\; & 2\; & 2\; & 2\; & 1\; & 1 & 1 & 2 & 2 & 2\\ 2\; & 0\; & 2\; & 2\; & 1\; & 2 & 2 & 1 & 1 & 2\\ 2\; & 2\; & 0\; & 2\; & 2\; & 1 & 2 & 1 & 2 & 1\\ 2\; & 2\; & 2\; & 0\; & 2\; & 2 & 1 & 2 & 1 & 1\\ 1\; & 1\; & 2\; & 2\; & 2\phi \; & 1+\psi & 1+\psi & 1+\psi & 1+\psi & 2\\ 1\; & 2\; & 1\; & 2\; & 1+\psi \; & 2\phi & 1+\psi & 1+\psi & 2 & 1+\psi \\ 1\; & 2\; & 2\; & 1\; & 1+\psi \; & 1+\psi & 2\phi & 2 & 1+\psi & 1+\psi \\ 2\; & 1\; & 1\; & 2\; & 1+\psi \; & 1+\psi & 2 & 2\phi & 1+\psi & 1+\psi \\ 2\; & 1\; & 2\; & 1\; & 1+\psi \; & 2 & 1+\psi & 1+\psi & 2\phi & 1+\psi \\ 2\; & 2\; & 1\; & 1\; & 2\; & 1+\psi & 1+\psi & 1+\psi & 1+\psi & 2\phi \end{array}\right], \end{array}$$

where

(3) $$\begin{array}{c} \phi =\frac{r^{2}}{10r^{2}-18r+9},\\ \psi =\frac{r}{3-2r}. \end{array}$$

Based on matrices (1) and (2), the expressions for the frequencies of double reduction (*α* and *β*) and the recombination fraction *r* can be expressed in terms of *g*_*i*_ as

(4) $$\begin{array}{c} \alpha =g_{1}+g_{2}+g_{3}+g_{4},\\ \beta =g_{1}+g_{2}+g_{5}+g_{6},\\ r=\frac{1}{2}\left[g_{3}+g_{5}+2\left(g_{2}+g_{4}+g_{6}+g_{9}\right)+2\phi g_{7}+\left(1+\psi \right)g_{8}\right]. \end{array}$$

### 2.3. Quantitative Genetic Model

For a given QTL, there are 10 different QTL gamete genotypes in the multivalent tetraploid, whose values can be partitioned into additive and dominance genetic effects of different types, expressed as

(5) $$\begin{array}{cc} & \mu_{11}=\mu +a_{1},\;\text{for}\;Q_{1}Q_{1},\\ & \mu_{22}=\mu +a_{2},\;\text{for}\;Q_{2}Q_{2},\\ & \mu_{33}=\mu +a_{3},\;\text{for}\;Q_{3}Q_{3},\\ & \mu_{44}=\mu -a_{1}-a_{2}-a_{3},\;\text{for}\;Q_{4}Q_{4},\\ & \mu_{12}=\mu +a_{1}+a_{2}+d_{12},\;\text{for}\;Q_{1}Q_{2},\\ & \mu_{13}=\mu +a_{1}+a_{3}+d_{13},\;\text{for}\;Q_{1}Q_{3},\\ & \mu_{14}=\mu -a_{2}-a_{3}+d_{14},\;\text{for}\;Q_{1}Q_{4},\\ & \mu_{23}=\mu +a_{2}+a_{3}+d_{23},\;\text{for}\;Q_{2}Q_{3},\\ & \mu_{24}=\mu -a_{1}-a_{3}+d_{24},\;\text{for}\;Q_{2}Q_{4},\\ & \mu_{34}=\mu -a_{1}-a_{2}+d_{34},\;\text{for}\;Q_{3}Q_{4}, \end{array}$$

where  *μ* is the overall mean, *a*_1_, *a*_2_, and *a*_3_ are the additive genetic effects of alleles *Q*_1_, *Q*_2_, and *Q*_3_ relative to allele *Q*_4_, and *d*_12_, *d*_13_, *d*_14_, *d*_23_, *d*_24_, and *d*_34_ are the dominant genetic effects due to interactions between different alleles *Q*_1_ and *Q*_2_, *Q*_1_ and *Q*_3_, *Q*_1_ and *Q*_4_, *Q*_2_ and *Q*_3_, *Q*_2_ and *Q*_4_, and *Q*_3_ and *Q*_4_, respectively.

From expression (5), we can solve the overall mean and additive and dominant effects as

(6) $$\begin{array}{c} \mu =\frac{1}{4}\left(\mu_{11}+\mu_{22}+\mu_{33}+\mu_{44}\right), \end{array}$$

(7) $$\begin{array}{c} a_{1}=\frac{1}{4}\left(3\mu_{11}-\mu_{22}-\mu_{33}-\mu_{44}\right), \end{array}$$

(8) $$\begin{array}{c} a_{2}=\frac{1}{4}\left(3\mu_{22}-\mu_{11}-\mu_{33}-\mu_{44}\right), \end{array}$$

(9) $$\begin{array}{c} a_{3}=\frac{1}{4}\left(3\mu_{33}-\mu_{11}-\mu_{22}-\mu_{44}\right), \end{array}$$

(10) $$\begin{array}{c} d_{12}=\mu_{12}-\frac{1}{4}\left(3\mu_{33}+3\mu_{44}-\mu_{11}-\mu_{22}\right), \end{array}$$

(11) $$\begin{array}{c} d_{13}=\mu_{13}-\frac{1}{4}\left(3\mu_{22}+3\mu_{44}-\mu_{11}-\mu_{33}\right), \end{array}$$

(12) $$\begin{array}{c} d_{14}=\mu_{14}-\frac{1}{4}\left(3\mu_{22}+3\mu_{33}-\mu_{11}-\mu_{44}\right), \end{array}$$

(13) $$\begin{array}{c} d_{23}=\mu_{23}-\frac{1}{4}\left(3\mu_{11}+3\mu_{44}-\mu_{22}-\mu_{33}\right), \end{array}$$

(14) $$\begin{array}{c} d_{24}=\mu_{24}-\frac{1}{4}\left(3\mu_{11}+3\mu_{33}-\mu_{22}-\mu_{44}\right), \end{array}$$

(15) $$\begin{array}{c} d_{34}=\mu_{34}-\frac{1}{4}\left(3\mu_{11}+3\mu_{22}-\mu_{33}-\mu_{44}\right). \end{array}$$

### 2.4. EM Algorithm

Ignoring the effects of other covariates, the phenotypic value, *y*_*i*_, for individual *i* in the pseudotest backcross can be expressed in terms of the QTL effect and residual error as

(16) $$\begin{array}{c} y_{i}=\overset{4}{\underset{j_{1}\leq j_{2}=1}{\sum }}\xi_{j_{1}j_{2}\;|\;i}\mu_{j_{1}j_{2}}+e_{i}, \end{array}$$

where *ξ*_*j*_1_*j*_2_∣*i*_ is the indicator variable that is defined as 1 if individual *i* has a QTL genotype *j*_1_ *j*_2_ (*j*_1_ ≤ *j*_2_ = *Q*_1_, *Q*_2_, *Q*_3_, *Q*_4_), and 0 otherwise, *μ*_*j*_1_*j*_2__ is the genotypic value of QTL genotype *j*_1_ *j*_2_ as defined in (5), and *e*_*i*_ is the residual error assumed to be normally distributed with mean zero and variance *σ* ^2^. We use **Ω** to denote the unknown vector (*μ*_11_, *μ*_22_, *μ*_33_, *μ*_44_, *μ*_12_, *μ*_13_, *μ*_14_, *μ*_23_, *μ*_24_, *μ*_34_, *σ* ^2^).

For a QTL mapping experiment, marker genotypes are observable. Let *n*_*l*_1_*l*_2__ be the observation of marker genotype *l*_1_ *l*_2_ (*l*_1_ ≤ *l*_2_ = *M*_1_, *M*_2_, *M*_3_, *M*_4_). The likelihood of the phenotypic (**y**) and marker data (**M**) is constructed, within the mixture model framework, as

(17) $$\begin{array}{cc} L\left(\Omega \;|\;y,M\right) & =\overset{n}{\underset{i=1}{\prod }}\;\overset{M_{4}}{\underset{l_{1}=M_{1}}{\prod }}\;\overset{M_{4}}{\underset{l_{2}=M_{1}}{\prod }}\;\overset{Q_{4}}{\underset{j_{1}=Q_{1}}{\sum }}\;\overset{Q_{4}}{\underset{j_{2}=Q_{1}}{\sum }}\pi_{j_{1}j_{2}\;|\;l_{1}l_{2}}f_{j_{1}j_{2}}\left(y_{i}\right),\\ & \;\;\;\;\;\;\;\;\;\;\;l_{1}\leq l_{2},j_{1}\leq j_{2}, \end{array}$$

where *π*_*j*_1_*j*_2_∣*l*_1_*l*_2__ is the conditional probability of QTL genotype *j*_1_ *j*_2_ given marker genotype *l*_1_ *l*_2_, and *f*_*j*_1_*j*_2__(*y*_*i*_) is assumed to follow a normal distribution with mean *μ*_*j*_1_*j*_2__ and variance *σ* ^2^. Prior conditional probability *π*_*j*_1_*j*_2_∣*l*_1_*l*_2__ is calculated as the frequency of joint marker-QTL genotype *l*_1_ *l*_2_ *j*_1_ *j*_2_, expressed in terms of nine *g* probabilities in matrix (1), divided by the frequency of marker genotype *l*_1_ *l*_2_. Marker genotype frequencies are *α*/4 for each of double reduction gametes *M*_1_ *M*_1_, *M*_2_ *M*_2_, *M*_3_ *M*_3_, and *M*_4_ *M*_4_, and (1 − *α*)/6 for each of nondouble reduction gametes *M*_1_ *M*_2_, *M*_1_ *M*_3_, *M*_1_ *M*_4_, *M*_2_ *M*_3_, *M*_2_ *M*_4_, and *M*_3_ *M*_4_.

The estimates of unknown parameters that maximize the likelihood (17) can be obtained by implementing the EM algorithm. In step E, we calculate the posterior probability of a QTL genotype given a specific marker genotype of individual *i* by

(18) $$\begin{array}{c} \Theta_{j_{1}j_{2}\;|\;l_{1}l_{2}i}=\frac{\pi_{j_{1}j_{2}\;|\;l_{1}l_{2}}f_{j_{1}j_{2}}\left(y_{i}\right)}{\sum_{j_{1}^{\prime }=Q_{1}}^{Q_{4}}\sum_{j_{2}^{\prime }=Q_{1}}^{Q_{4}}\pi_{j_{2}^{1}j_{2}^{\prime }\;|\;l_{1}l_{2}}f_{j_{1}^{\prime }j_{2}^{\prime }}\left(y_{i}\right)}. \end{array}$$

In step M, we calculate the frequencies of nine observable gamete modes based on the calculated posterior probabilities using the following: 


(19)
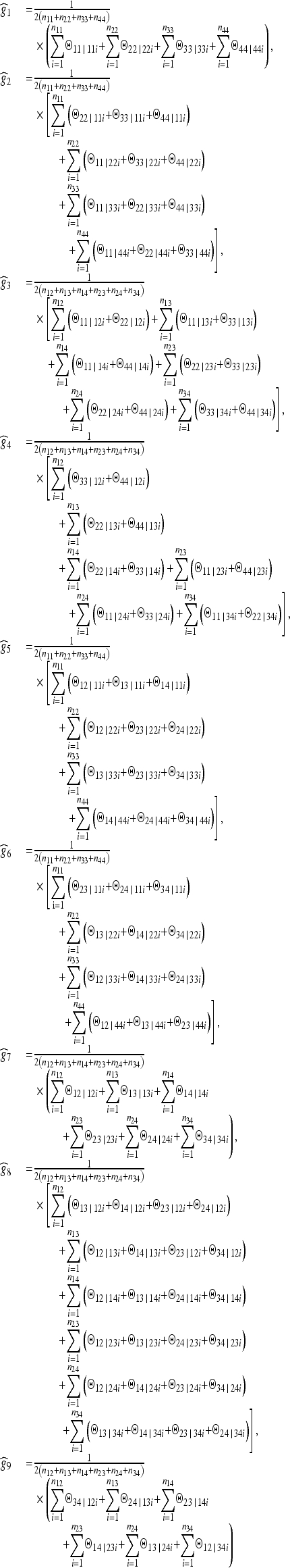

which lead to the estimates of the frequencies of double reduction as

(20) $$\begin{array}{cc} \hat{\alpha } & ={\hat{g}}_{1}+{\hat{g}}_{2}+{\hat{g}}_{3}+{\hat{g}}_{4}\\ & =\frac{1}{n}\left(n_{11}+n_{22}+n_{33}+n_{44}\right),\\ \hat{\beta } & ={\hat{g}}_{1}+{\hat{g}}_{2}+{\hat{g}}_{5}+{\hat{g}}_{6},\\ \hat{r} & =\frac{1}{2}[{\hat{g}}_{3}+{\hat{g}}_{5}+2\left({\hat{g}}_{2}+{\hat{g}}_{4}+{\hat{g}}_{6}+{\hat{g}}_{9}\right)\;\\ & \;\;\;+2\phi {\hat{g}}_{7}+\left(1+\psi \right){\hat{g}}_{8}]. \end{array}$$

The genotypic value of QTL genotype *j*_1_ *j*_2_ and residual variance are estimated by

(21) $$\begin{array}{cc} {\hat{\mu }}_{j_{1}j_{2}} & =\frac{\sum_{\text{i}=1}^{n}\sum_{l_{1}=M_{1}}^{M_{4}}\sum_{l_{2}=M_{1}}^{M_{4}}\Theta_{j_{1}j_{1}\;|\;l_{1}l_{2}i}y_{i}}{\sum_{l_{1}=M_{1}}^{M_{4}}\sum_{l_{2}=M_{1}}^{M_{4}}\Theta_{j_{1}j_{1}\;|\;l_{1}l_{2}i}},\\ & \;\;\;\;\;\;\;\;l_{1}\leq l_{2};j_{1}\leq j_{2}, \end{array}$$

(22) $$\begin{array}{cc} {\hat{\sigma }}^{2} & =\frac{1}{n}\overset{n}{\underset{i=1}{\sum }}\;\overset{M_{4}}{\underset{l_{1}=M_{1}}{\sum }}\;\overset{M_{4}}{\underset{l_{2}=M_{1}}{\sum }}\;\overset{Q_{4}}{\underset{j_{1}=Q_{1}}{\sum }}\;\overset{Q_{4}}{\underset{j_{2}=Q_{1}}{\sum }}\Theta_{j_{1}j_{1}\;|\;l_{1}l_{2}i}{\left(y_{i}-{\hat{\mu }}_{j_{1}j_{2}}\right)}^{2},\\ & \;\;\;\;\;\;\;\;\;\;\;\;\;\;l_{1}\leq l_{2};j_{1}\leq j_{2}. \end{array}$$

The iteration is repeated between the E step, (3) and (18), and M step, (19)–(22), until stable estimates are obtained. The stable estimates are the maximum likelihood estimates (MLEs) of parameters.

### 2.5. Hypothesis Testing

 Following parameter estimation, several hypotheses should be tested. The hypothesis about the presence of a QTL segregating in the pseudotest backcrosses is formulated as

(23) $$\begin{array}{c} H_{0}\text{:}\;a_{1}=a_{2}=a_{3}=d_{12}=d_{13}=d_{14}=d_{23}=d_{24}=d_{34}=0,\\ H_{1}\text{:}\;\text{at}\;\text{least}\;\text{one}\;\text{of}\;\text{them}\;\text{is}\;\text{not}\;\text{equal}\;\text{to}\;\text{zero}. \end{array}$$

The difference between the log-likelihood functions under the null and alternative hypotheses are calculated. But the distribution of this log-likelihood ratio (LR) is not known because of the violation of regularity conditions for the mixture model (1). For this reason, a commonly used empirical approach based on permutation tests by reshuffling the relationships between the marker genotypes and phenotypes [26] is used to determine the critical threshold, in order to judge whether there is a QTL for the trait.

After a significant QTL is detected, the next hypothesis is about the additive genetic effect of the QTL. This can be tested by formulating the null hypothesis,

(24) $$\begin{array}{c} H_{0}\text{:}\;a_{1}=a_{2}=a_{3}=0, \end{array}$$

under which the estimates of genotypic values of QTL genotypes can be obtained with the EM algorithm as described above, but posing three constraints derived from (7), (8), and (9). Similarly, the dominant genetic effects can be tested with the null hypothesis,

(25) $$\begin{array}{c} H_{0}\text{:}\;d_{12}=d_{13}=d_{14}=d_{23}=d_{24}=d_{34}=0, \end{array}$$

with estimates of genotypic values under the constraints derived from (10)–(15). All these genetic effects can be tested individually.

### 2.6. Application to Simulated Data

A pseudotest backcross for a multivalent tetraploid was hypothesized, in which a marker is assumed to be linked with a QTL that affects a quantitative trait. Marker and QTL genotypes were simulated for the pseudotest backcross of different sample sizes (*n* = 100,200,400) based on a range of double reduction (0.05, 0.15, 0.30) and recombination fraction (0.05, 0.25). We assume the same frequency of double reduction between the marker and QTL. The phenotypic value of an individual is expressed as the summation of genotypic values of a QTL genotype carried by this individual and a normally distributed error. The genotypic values of a QTL genotype are calculated by (5), where the overall mean  *μ* is assigned as 1, and the additive and dominant effects assigned as *a*_1_ = *a*_2_ = *a*_3_ = 0.6 and *d*_12_ = *d*_13_ = *d*_14_ = *d*_23_ = *d*_24_ = *d*_34_ = 0.5. The error variance is determined according to the heritability of *H* ^2^ = 0.1 and  0.4, respectively.

In this simulation study, fully informative markers and QTL are assumed and, thus, the double reduction at the marker can be estimated analytically. The estimates of the parameters converge to stable values at a rapid rate given that there are closed forms for parameter estimators in the EM framework. We evaluate the estimation of the other parameters related to QTL segregation, effects, and position. The means of the MLEs of the QTL-related parameters and their standard errors based on 1000 simulation replicates are illustrated in Tables 1, 2, and 3. With a small sample size (100), the double reduction of the QTL was accurately estimated, with the precision of estimation relatively independent of the magnitude of heritability and the degree of QTL-marker linkage (Table 1). The most significant factor that affected the estimate of QTL position (in terms of its recombination with the marker) was the heritability, followed by sample size and the degree of QTL-marker linkage. In general, at least a sample size of 200 was required to reliably estimate the QTL position for a major gene that explains about 20%–30% of the phenotypic variance.

The estimation precision of the QTL effects depended on the heritability, sample size, and degree of QTL-marker linkage. As heritability, sample size, and linkage degree increased, the estimates of various QTL effects were more precise. As compared with the dominant genetic effects, the estimates of the additive genetic effects required a larger sample size, more precise phenotypic measurements (leading to a higher heritability), and a denser linkage map (with a stronger degree of QTL-marker linkage). We found that the estimates of QTL effects were influenced by the frequency of QTL double reduction. At low frequencies of double reduction, the effects of QTL were more accurately estimated than at higher frequencies. For a QTL undergoing a strong double reduction (say *α* = 0.3), a sample size of at least 400 is required even if the QTL explains a large proportion of the phenotypic variance (0.4). For a modest-sized QTL, a much larger sample size was required.

We performed a simulation study to test how the misspecification of double reduction affects the estimate of QTL-related parameters. This was done by using traditional mapping models (without considering double reduction) to analyze the simulated data of QTL genotypes with different degrees of double reduction. When a QTL undergoes double reduction, traditional models that do not consider double reduction provided misleading results about the estimates of QTL effects and position (data not shown). Furthermore, increasing heritabilities and sample sizes did not improve the estimates. In this case, the power of QTL detection was reduced.

## 3. Discussion

A statistical method for genetic mapping of quantitative trait loci (QTLs) in a multivalent tetraploid undergoing a double reduction process is described. As an important cytological characteristic of polyploids, double reduction may play a significant role in plant evolution and maintenance of genetic polymorphism in natural populations. Also, because double reduction affects the result of linkage analysis through the crossing-over events between different chromosomes [24, 25], it is important to incorporate double reduction into a QTL mapping framework. This method provides a powerful tool for QTL mapping and understanding the genetic control of a quantitative trait in a multivalent tetraploid.

The method capitalizes on 11 different classifications of two-locus gamete formations, derived by Fisher [23], during multivalent tetraploid meiosis and has proven to be powerful for simultaneous estimation of the frequencies of double reduction and the recombination fraction between different loci. Although a couple of statistical approaches have been proposed to map multivalent tetraploid QTLs [26, 27], this method has for the first time incorporated Fisher's tetrasomic inheritance into the mapping framework, thus enhancing the cytological relevance of QTL detection. Results from simulation studies showed that the method can be used to map QTLs in a controlled cross of multivalent tetraploids when the mapping population is adequately large (say 400). When a QTL undergoes double reduction, traditional mapping approaches will incorrectly estimate the position and effects of the QTL, proportional to the degree of double reduction. The new method can estimate the double reduction of a QTL, an important parameter related to the genetic diversity and evolution of polyploids [28, 29].

Because of the high complexity of the mixture model implemented with tetrasomic inheritance, we only considered a one-marker model for QTL mapping. Interval mapping, which localizes a QTL with two flanking markers, has proven to be more advantageous in parameter estimation over the one-marker model [30]. It will be worthwhile to integrate components of our model into the interval mapping framework to fully explore the statistical merits of interval mapping for QTL mapping in multivalent tetraploids. Furthermore, the model proposed in this article assumes the segregation of fully informative codominant loci, each with 10 distinct genotypes, in a controlled cross of multivalent tetraploids. For partially informative codominant markers, a two-stage hierarchical mixture model will be needed to model the different allelic configurations for a phenotypically identical genotype. Although molecular marker technologies have improved in recent years, dominant markers may still be used in genetic mapping projects of some underrepresentative species including polyploids. Thus, it is also important to extend our model to map QTLs with dominant markers. For partially informative loci, the number of QTL genotypes may be unknown and, thus, a model selection procedure should be incorporated to determine the optimal number of genotypes at a QTL.

The genetic mapping of polyploids is complex because of their complex inheritance modes. Sophisticated statistical models are required to tackle genetic problems hidden in the polysomic inheritance of polyploids. Currently, there are some debates on the optimal modeling of tetrasomic inheritance in linkage analysis [13, 25] and QTL mapping [18, 31] partly because of our limited knowledge about these fascinating species. Before a detailed understanding of the cytological mechanisms for meioses in multivalent polyploids is obtained, this type of debate will continue. In any case, the development of powerful statistical models for polyploid mapping continues to be a pressing need. The application of these models to real-world data will not only test their usefulness, but also provide an unprecedented opportunity to understand the genetic differentiation among polyploid genomes and characterize the genetic architecture of quantitatively inherited traits for this unique group of species. Software for the method described is available at http://statgen.psu.edu/.

## Figures and Tables

**Table 1:** Maximum likelihood estimates of the double reduction, recombination fraction, overall mean, additive effects, and dominant effects under different simulation scenarios in a mapping population of size 100. Numbers in parentheses are the standard errors of the estimates.

True value					*μ*	*a*_1_	*a*_2_	*a*_3_	*d*_12_	*d*_13_	*d*_14_	*d*_23_	*d*_24_	*d*_34_
*α*	*r*	*H* ^2^	$\hat{\alpha }$	$\hat{r}$	1	0.6	0.6	0.6	0.5	0.5	0.5	0.5	0.5	0.5
0.3	0.25	0.1	0.295	0.314	1.153	0.047	0.116	0.037	2.228	3.007	−1.953	2.375	−2.661	−2.107
			(0.047)	(0.120)	(1.139)	(2.339)	(2.651)	(2.496)	(4.013)	(4.237)	(4.499)	(4.349)	(4.453)	(4.407)
0.3	0.25	0.4	0.301	0.251	1.095	0.556	0.496	0.546	0.677	0.625	0.313	0.482	0.181	0.138
			(0.048)	(0.098)	(0.540)	(0.986)	(1.003)	(1.133)	(1.744)	(1.861)	(2.143)	(1.947)	(2.103)	(1.931)
0.3	0.05	0.1	0.297	0.142	0.961	0.659	0.580	0.512	1.216	1.130	−0.036	1.286	0.119	−0.084
			(0.041)	(0.114)	(0.767)	(1.330)	(1.293)	(1.303)	(1.967)	(2.208)	(2.301)	(2.129)	(2.193)	(2.102)
0.3	0.05	0.4	0.296	0.057	0.998	0.642	0.643	0.533	0.445	0.562	0.428	0.601	0.488	0.594
			(0.045)	(0.051)	(0.296)	(0.461)	(0.581)	(0.497)	(0.868)	(0.787)	(0.851)	(0.973)	(0.762)	(0.687)

0.15	0.25	0.1	0.150	0.305	1.058	0.244	0.821	0.945	1.232	1.334	0.167	0.660	−0.150	−0.373
			(0.034)	(0.155)	(1.067)	(1.800)	(1.794)	(1.965)	(2.707)	(2.645)	(2.819)	(2.820)	(2.658)	(2.614)
0.15	0.25	0.4	0.152	0.239	1.061	0.522	0.548	0.598	0.645	0.484	0.449	0.492	0.382	0.230
			(0.031)	(0.097)	(0.369)	(0.777)	(0.812)	(0.794)	(1.082)	(1.166)	(1.100)	(1.203)	(1.227)	(1.238)
0.15	0.05	0.1	0.144	0.168	0.837	0.411	0.540	0.930	1.616	1.242	0.271	1.033	−0.037	−0.313
			(0.035)	(0.127)	(1.108)	(2.103)	(1.962)	(1.635)	(2.854)	(2.698)	(2.488)	(2.452)	(3.147)	(2.700)
0.15	0.05	0.4	0.143	0.057	0.921	0.372	0.736	0.545	0.685	0.965	0.621	0.677	0.282	0.453
			(0.035)	(0.058)	(0.488)	(0.877)	(0.759)	(0.775)	(1.192)	(1.148)	(1.085)	(1.085)	(1.167)	(1.147)

0.05	0.25	0.1	0.052	0.371	0.769	0.356	0.300	0.455	2.239	2.178	−0.691	2.031	−0.426	−0.588
			(0.022)	(0.180)	(1.293)	(2.122)	(2.576)	(2.392)	(3.425)	(3.478)	(3.104)	(2.787)	(2.998)	(3.563)
0.05	0.25	0.4	0.051	0.253	0.643	0.440	0.406	0.458	1.243	1.202	0.500	1.224	0.553	0.362
			(0.022)	(0.111)	(0.699)	(1.067)	(1.003)	(1.105)	(1.522)	(1.605)	(1.341)	(1.541)	(1.483)	(1.482)
0.05	0.05	0.1	0.052	0.193	1.008	0.056	1.012	0.362	1.552	1.977	−0.065	1.068	−1.077	−0.438
			(0.025)	(0.136)	(1.346)	(2.119)	(2.537)	(2.121)	(3.110)	(2.718)	(3.191)	(3.427)	(3.354)	(3.391)
0.05	0.05	0.4	0.052	0.064	0.762	0.525	0.361	0.337	1.119	1.175	0.193	1.255	0.303	0.351
			(0.024)	(0.059)	(0.736)	(1.005)	(0.959)	(1.076)	(1.480)	(1.482)	(1.542)	(1.636)	(1.473)	(1.285)

**Table 2:** Maximum likelihood estimates of the double reduction, recombination fraction, overall mean, additive effects, and dominant effects under different simulation scenarios in a mapping population of size 200. Numbers in parentheses are the standard errors of the estimates.

True value					*μ*	*a*_1_	*a*_2_	*a*_3_	*d*_12_	*d*_13_	*d*_14_	*d*_23_	*d*_24_	*d*_34_
*α*	*r*	*H* ^2^	$\hat{\alpha }$	$\hat{r}$	1	0.6	0.6	0.6	0.5	0.5	0.5	0.5	0.5	0.5
0.3	0.25	0.1	0.302	0.313	1.181	0.397	0.218	0.126	2.303	2.553	−1.649	2.272	−1.471	−1.268
			(0.031)	(0.093)	(1.207)	(1.708)	(1.697)	(1.443)	(2.844)	(3.144)	(3.576)	(3.048)	(3.454)	(3.526)
0.3	0.25	0.4	0.301	0.234	1.091	0.582	0.553	0.677	0.493	0.317	0.340	0.646	0.459	0.262
			(0.034)	(0.085)	(0.508)	(0.781)	(0.730)	(0.727)	(1.432)	(1.402)	(1.438)	(1.416)	(1.435)	(1.362)
0.3	0.05	0.1	0.302	0.093	1.013	0.545	0.551	0.660	0.739	0.703	0.206	0.924	0.114	0.089
			(0.032)	(0.093)	(0.483)	(0.789)	(0.829)	(0.753)	(1.432)	(1.561)	(1.309)	(1.355)	(1.453)	(1.486)
0.3	0.05	0.4	0.297	0.047	0.999	0.596	0.585	0.574	0.522	0.531	0.464	0.509	0.420	0.530
			(0.032)	(0.037)	(0.190)	(0.362)	(0.346)	(0.342)	(0.570)	(0.645)	(0.666)	(0.611)	(0.577)	(0.569)

0.15	0.25	0.1	0.152	0.277	1.025	0.692	0.563	0.587	0.953	0.968	−0.232	1.224	−0.034	−0.033
			(0.028)	(0.117)	(0.718)	(1.168)	(1.225)	(1.215)	(1.866)	(1.930)	(1.862)	(1.900)	(1.780)	(1.692)
0.15	0.25	0.4	0.147	0.234	0.982	0.581	0.675	0.580	0.420	0.551	0.561	0.386	0.484	0.617
			(0.024)	(0.078)	(0.287)	(0.520)	(0.471)	(0.530)	(0.838)	(0.724)	(0.775)	(0.822)	(0.745)	(0.736)
0.15	0.05	0.1	0.149	0.140	0.971	0.475	0.548	0.723	1.232	0.985	0.246	0.863	0.048	−0.325
			(0.024)	(0.106)	(0.633)	(1.251)	(1.257)	(1.479)	(1.814)	(1.719)	(1.990)	(1.968)	(1.868)	(1.824)
0.15	0.05	0.4	0.149	0.055	1.026	0.691	0.573	0.558	0.394	0.380	0.478	0.536	0.485	0.523
			(0.026)	(0.047)	(0.299)	(0.558)	(0.511)	(0.585)	(0.684)	(0.799)	(0.800)	(0.862)	(0.754)	(0.745)

0.05	0.25	0.1	0.049	0.326	1.074	0.750	0.325	0.557	1.235	1.190	−0.420	1.392	−0.127	−0.331
			(0.015)	(0.143)	(1.209)	(2.486)	(2.232)	(1.868)	(2.857)	(2.619)	(3.332)	(3.113)	(3.023)	(2.775)
0.05	0.25	0.4	0.048	0.241	0.932	0.619	0.345	0.528	0.885	0.658	0.234	0.879	0.498	0.242
			(0.013)	(0.075)	(0.499)	(1.038)	(1.037)	(0.932)	(1.365)	(1.305)	(1.282)	(1.317)	(1.190)	(1.276)
0.05	0.05	0.1	0.050	0.196	0.914	0.490	0.294	0.612	1.755	1.435	−0.487	1.713	−0.146	−0.379
			(0.015)	(0.127)	(1.209)	(1.941)	(2.134)	(2.157)	(2.902)	(2.792)	(2.747)	(2.872)	(2.758)	(2.855)
0.05	0.05	0.4	0.049	0.059	0.818	0.609	0.351	0.697	0.952	0.646	0.548	0.908	0.737	0.412
			(0.017)	(0.049)	(0.541)	(0.978)	(0.988)	(0.967)	(1.130)	(1.527)	(1.419)	(1.285)	(1.279)	(1.001)

**Table 3:** Maximum likelihood estimates of the double reduction, recombination fraction, overall mean, additive effects, and dominant effects under different simulation scenarios in a mapping population of size 400. Numbers in parentheses are the standard errors of the estimates.

True value					*μ*	*a*_1_	*a*_2_	*a*_3_	*d*_12_	*d*_13_	*d*_14_	*d*_23_	*d*_24_	*d*_34_
*α*	*r*	*H* ^2^	$\hat{\alpha }$	$\hat{r}$	1	0.6	0.6	0.6	0.5	0.5	0.5	0.5	0.5	0.5
0.3	0.25	0.1	0.300	0.300	1.208	0.182	0.435	0.208	1.800	2.002	−1.236	1.546	−1.574	−1.318
			(0.023)	(0.082)	(0.933)	(1.405)	(1.400)	(1.074)	(2.357)	(2.429)	(2.829)	(2.130)	(2.744)	(2.906)
0.3	0.25	0.4	0.301	0.231	1.066	0.585	0.557	0.603	0.508	0.451	0.361	0.494	0.463	0.309
			(0.023)	(0.078)	(0.358)	(0.552)	(0.497)	(0.532)	(1.050)	(0.984)	(0.977)	(1.115)	(1.060)	(0.964)
0.3	0.05	0.1	0.298	0.097	1.026	0.577	0.646	0.629	0.704	0.818	0.245	0.689	0.202	0.208
			(0.023)	(0.077)	(0.389)	(0.603)	(0.637)	(0.580)	(1.067)	(1.138)	(1.206)	(1.028)	(1.120)	(1.071)
0.3	0.05	0.4	0.299	0.050	1.014	0.626	0.553	0.639	0.525	0.442	0.458	0.507	0.575	0.414
			(0.025)	(0.027)	(0.127)	(0.245)	(0.253)	(0.250)	(0.388)	(0.435)	(0.405)	(0.403)	(0.388)	(0.350)

0.15	0.25	0.1	0.150	0.261	0.928	0.641	0.463	0.611	0.975	0.860	0.192	0.995	0.265	0.202
			(0.016)	(0.135)	(0.510)	(0.684)	(0.904)	(0.737)	(1.372)	(1.476)	(1.423)	(1.286)	(1.458)	(1.353)
0.15	0.25	0.4	0.150	0.250	1.012	0.584	0.624	0.565	0.495	0.552	0.479	0.516	0.452	0.424
			(0.017)	(0.060)	(0.180)	(0.329)	(0.358)	(0.326)	(0.511)	(0.526)	(0.510)	(0.558)	(0.478)	(0.419)
0.15	0.05	0.1	0.150	0.113	1.075	0.583	0.583	0.706	0.740	0.705	0.218	0.667	0.170	0.058
			(0.017)	(0.095)	(0.485)	(0.866)	(0.846)	(0.838)	(1.341)	(1.254)	(1.322)	(1.527)	(1.272)	(1.218)
0.15	0.05	0.4	0.150	0.049	1.014	0.526	0.654	0.607	0.531	0.572	0.547	0.406	0.425	0.482
			(0.017)	(0.034)	(0.197)	(0.307)	(0.382)	(0.366)	(0.557)	(0.511)	(0.551)	(0.516)	(0.506)	(0.483)

0.05	0.25	0.1	0.050	0.322	0.949	0.516	0.726	0.629	1.256	1.327	0.187	1.026	−0.191	−0.037
			(0.010)	(0.113)	(0.858)	(1.819)	(1.626)	(1.773)	(2.170)	(2.180)	(2.155)	(2.213)	(2.437)	(2.459)
0.05	0.25	0.4	0.050	0.250	1.007	0.610	0.652	0.551	0.404	0.562	0.481	0.480	0.457	0.545
			(0.012)	(0.057)	(0.423)	(0.664)	(0.680)	(0.624)	(0.873)	(0.789)	(0.867)	(0.916)	(0.909)	(0.991)
0.05	0.05	0.1	0.050	0.167	1.002	0.656	0.343	0.723	1.156	0.796	−0.164	1.147	0.111	−0.211
			(0.011)	(0.109)	(0.830)	(1.548)	(1.553)	(1.581)	(2.032)	(2.084)	(2.157)	(2.213)	(1.997)	(2.140)
0.05	0.05	0.4	0.051	0.054	1.025	0.591	0.608	0.635	0.512	0.459	0.518	0.469	0.477	0.450
			(0.011)	(0.037)	(0.347)	(0.637)	(0.624)	(0.698)	(0.771)	(0.895)	(0.894)	(0.931)	(0.800)	(0.770)
